# Sagittal and Vertical Growth of the Maxillo–Mandibular Complex in Untreated Children: A Longitudinal Study on Lateral Cephalograms Derived from Cone Beam Computed Tomography

**DOI:** 10.3390/s21248484

**Published:** 2021-12-20

**Authors:** Leah Yi, Hyeran Helen Jeon, Chenshuang Li, Normand Boucher, Chun-Hsi Chung

**Affiliations:** Department of Orthodontics, School of Dental Medicine, University of Pennsylvania, Philadelphia, PA 19104, USA; leahyi@upenn.edu (L.Y.); hjeon@upenn.edu (H.H.J.); lichens@upenn.edu (C.L.); nboucher@upenn.edu (N.B.)

**Keywords:** sagittal growth, vertical growth, longitudinal study, CBCT, untreated

## Abstract

The aim of this longitudinal study was to evaluate the sagittal and vertical growth of the maxillo–mandibular complex in untreated children using orthogonal lateral cephalograms compressed from cone beam computed tomography (CBCT). Two sets of scans, on 12 males (mean 8.75 years at T1, and 11.52 years at T2) and 18 females (mean 9.09 years at T1, and 10.80 years at T2), were analyzed using Dolphin 3D imaging. The displacements of the landmarks and rotations of both jaws relative to the cranial base were measured using the cranial base, and the maxillary and mandibular core lines. From T1 to T2, relative to the cranial base, the nasion, orbitale, A-point, and B-point moved anteriorly and inferiorly. The porion moved posteriorly and inferiorly. The ANB and mandibular plane angle decreased. All but one subject had forward rotation in reference to the cranial base. The maxillary and mandibular superimpositions showed no sagittal change on the A-point and B-point. The U6 and U1 erupted at 0.94 and 1.01 mm/year (males) and 0.82 and 0.95 mm/year (females), respectively. The L6 and L1 erupted at 0.66 and 0.88 mm/year (males), and at 0.41 mm/year for both the L6 and the L1 (females), respectively.

## 1. Introduction

Orthodontic treatments involve orthopedic and dental effects in adolescent patients. Their natural facial growth also plays an important role in the final treatment outcomes for these patients. Hence, a proper diagnosis and treatment planning is highly dependent on a firm understanding of craniofacial growth and development.

Generally, there is a consensus that certain facial types can be characterized by unique growth patterns. Bjork and Skieller [1] used implants to show that the maxilla and mandible display a forward rotation in most children (19 out of 21), and a backward rotation in a few children (2 out of 21). The forward rotation of the mandible was accompanied by the forward growth of the condyles, and bone resorption on the lower border of the ramus, whereas the backward rotation of the mandible showed the backward growth of the condyles, and bone apposition on the lower border of the ramus. On the maxilla, bone resorption occurred on the nasal floor, which would increase the volume of the nasal cavity.

Implant and anthropomorphic studies have identified which areas of the craniofacial complex are stable, maintaining a predictable relationship with each other throughout growth [1,2,3]. These stable craniofacial structures have been used to evaluate sagittal and vertical growth on lateral cephalograms. The anterior wall of the sella turcica, the greater wings of the sphenoid, the cribriform plate, the ethmoid crest, the planum sphenoidale, and the cerebral surfaces of the frontal bone have been shown to be stable, and are the current standards for cranial base superimposition [4,5,6,7,8,9]. To assess intramaxillary changes, the maxilla should be superimposed on the anterior surface of the zygomatic process and the maxilla/zygomatico/temporal sulci. A maxillary superimposition should demonstrate the downward and backward remodeling of the lower aspect of the key ridge, and the apposition and resorption of the orbital and nasal floors in a 3/5 to 2/5 ratio, respectively [2,10,11,12]. The point on the anterior surface of the chin just below the pogonion, the inner contour of the most inferior aspect of the symphysis, and the posterior contour of the inferior alveolar canal are considered the stable superimposition structures in the mandible [3,13,14,15,16,17,18].

The identification of stable landmarks by the implant studies of Bjork and Skieller [1,2,3] has enabled the further study of growth and development through their use as reference structures. Wang et al. used the stable landmarks identified in the mandible to study mandibular rotation in adolescents [17]. Similar to the findings of Bjork and Skieller, they observed forward mandibular rotation relative to the cranial base in adolescents, and compensatory backward rotation of the mandibular plane relative to the mandible. West et al. also used the stable craniofacial landmarks to observe the forward movement of the maxilla and mandible relative to the cranial base, from adolescence to adulthood [19]. Furthermore, they documented the eruption of the maxillary and mandibular central incisors and the first molars relative to the maxilla and mandible, respectively. Pinto et al. [20] identified condylar growth in the superior and posterior directions relative to the mandible reference structures, which is consistent with the findings in the implant studies of Bjork and Skieller [1,2,3], and Baurmind et al. [21].

Most of the literature on craniofacial growth is based on 2D radiography. However, magnification, the superimposition of structures, and variable head positioning limit the accuracy of 2D images analyses. These limitations can be overcome with 3D cone beam computed tomography (CBCT) images, which are compressed to produce orthogonal, or nonmagnified, 2D lateral cephalograms [22,23,24]. It has been reported that the orthogonal CBCT-synthesized cephalograms provided measurements closest to the actual skull measurements, and were significantly more precise than conventional cephalograms [22]. The 2D image can be compressed from the outer portion of one side of the head to the center of the central incisor area of the other side, into a 2D orthogonal projection, which increases the clarity of structures that are normally obscured by the superimposed contralateral side [24].

The purpose of this study was to conduct a longitudinal evaluation of craniofacial growth in the sagittal and vertical dimensions in untreated children using orthogonal lateral cephalograms derived from CBCT images.

## 2. Materials and Methods

This study was approved by the University of Pennsylvania International Review Board and Ethics Committee (#830045). All CBCT images were oriented and analyzed using Dolphin Imaging 3D software (version 11.9, Dolphin Imaging & Management Solutions, Chatsworth, CA, USA).

### 2.1. Sample

As we previously published [25], untreated subjects who had CBCT images taken at different periods were collected from various private practices utilizing CBCT imaging (i-CAT^TM^, KaVo Imaging, Hatfield, PA, USA) in routine record exams, from November 2006 to July 2016. These CBCT images were taken at 120 kVp and 5 mA, with a volume size of 16 × 13 cm^2^, a voxel size of 0.3 mm, and an exposure time of 3.7 s. The subjects were not treated at the initial exam (T1) for various reasons. For example, some patients were referred for ENT consultations because of hypertrophy of the adenoids and tonsils. Several sought second opinions, and others postponed their treatment for financial reasons, and then came back for the treatment (T2). The sample populations were comprised of 14 skeletal Cl I (ANB: 1°–4°) patients, 12 mild-to-moderate Cl II (ANB > 4°, 4.1–6.8) patients, due to deficient mandibles, and 4 mild Cl III (−0.5 < ANB < 1°) patients [26]. In addition, 26 patients had normal mandibular plane angles (SN-GoGn: 27°–36°), 1 had a slightly high mandibular angle (SN-GoGn: 38°), and 3 had low mandibular angles (SN-GoGn < 27°) [27]. The sample examined in this study included 12 males, who were an average age of 8.75 years old at T1 (5.4–11.48), and 11.52 years old at T2 (8.7–14.7), as well as 18 females, who were an average age of 9.09 years old at T1 (6.2–11.7), and 10.8 years old at T2 (7.2–13.7). None of the patients reported any craniofacial anomalies or significant medical conditions that might affect the growth and development of the craniofacial complex.

### 2.2. Orientation

**Figure 1:**
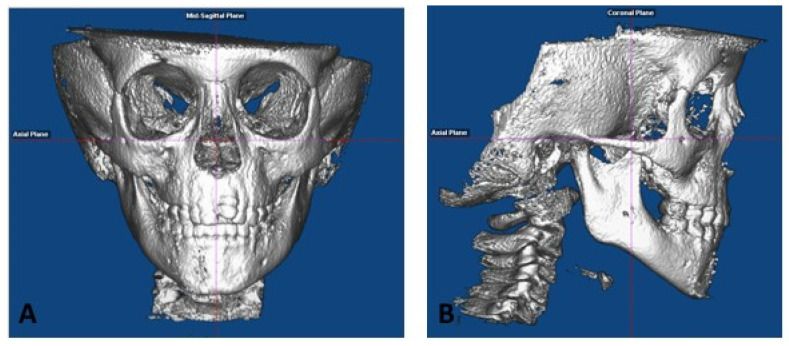
CBCT Orientation: (**A**) roll and yaw, and (**B**) pitch, as previously published in [25].

As we previously decribed [25]:

- Roll: The cranium was oriented such that a midline could be drawn through the midpoint of the frontonasal suture and the base of the nose, and was parallel to the true vertical, which is perpendicular to a true horizontal line (Figure 1A). A true horizontal line goes through the most inferior aspect of the orbits;
- Yaw: The cranium was oriented to achieve the best symmetry of the cranial base, zygomatic, and maxillary structures on either side of the midline (Figure 1A);
- Pitch: The cranium was oriented such that the Frankfurt horizontal plane was parallel to the true horizontal plane (Figure 1B).

### 2.3. Lateral Cephalograms Superimpositions and Construction of the “Core Lines”

**Figure 2:**
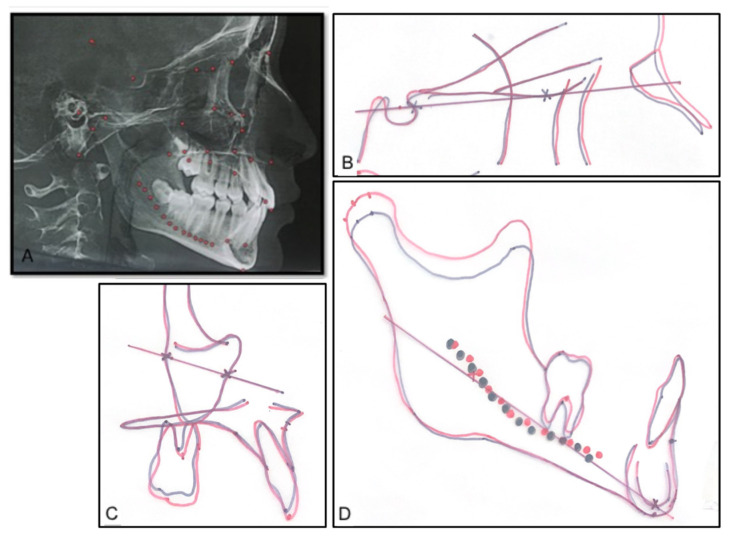
The cranial base, maxillary, and mandibular core lines. (**A**) Landmarks were placed on the CBCT image to facilitate the identification of certain structures. (**B**) The cranial base core line is drawn on the T1 lateral cephalograms and transferred onto the T2 tracing on a cranial base superimposition. (**C**) The maxillary core lines. (**D**) The mandibular core lines. Black: T1, and red: T2.

Lateral cephalograms were generated as an orthogonal view (0% magnification) of the right side of the face to the midpoint of the left maxillary central incisor, as previously described by Habeeb et al. [24]. Superimposition structures and landmarks were identified on the CBCT 3D image prior to the compression so that they could be clearly identified and used for the accurate superimposition of the T1 and T2 lateral cephalograms, as shown in Figure 2A.

**Figure 3:**
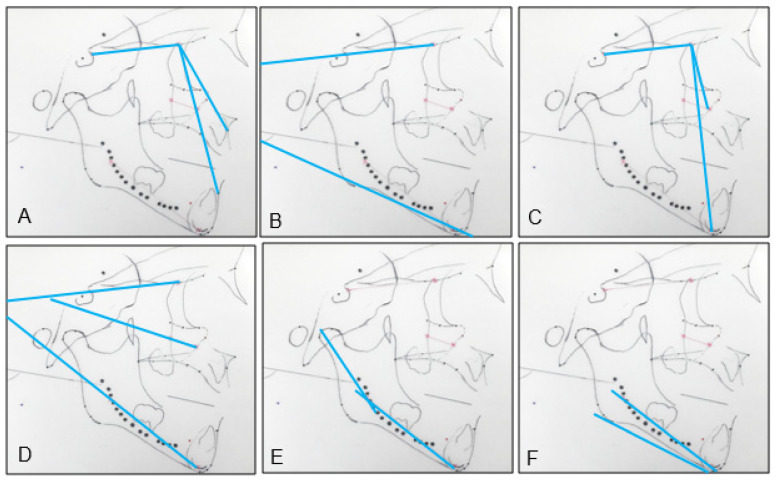
Angular measurements: (**A**) CB line-A, CB line-B, and A-CB point-B.; (**B**) CB line-GoGn.; (**C**) CB line-Mx point, CB line-Md point, and Mx point-CB line-Md point.; (**D**) CB line-Mx line and CB line-Md line; (**E**) Co-Md line.; (**F**) Md line-Md border.

All hand tracings were performed by one examiner (L.Y.), and were periodically calibrated with the senior faculty in the Department of Orthodontics at the University of Pennsylvania School of Dental Medicine. The tracings of the T1 and T2 lateral cephalograms were superimposed on the basis of the stable structures in the cranial base, maxilla, and mandible. On the cranial base, the tracings would be superimposed on the anterior wall of the sella turcica, the cribriform plate, the ethmoid crest, and the greater wing of the sphenoid. If more clarification was needed, the planum sphenoidale and the cerebral surfaces of the orbital roofs were used [28]. Fixed fiducial points were transferred from the T1 cephalometric tracing to the T2 tracing in the same subject (Figure 3B–D). This “cranial base core line (CB line)”, going through two points on the anterior wall of the sella turcica and on the cribriform plate, serves as a method by which the cranial base superimposition can be easily reproduced, and as a stable reference line from which various angular measurements can be made (Figure 2B). A “maxillary core line (Mx line)” was drawn between two points placed on the anterior surface of the zygoma and on the maxillary-zygomatico-temporal sulcus (Figure 2C). The maxillary tracings were superimposed on the anterior surface of the zygomatic process and the maxillary-zygomatico-temporal sulcus. The maxillary tracing was moved along the anterior surface of the zygomatic process so that the orbital floor of T2 is above that of T1, and so that the nasal floor of T2 is below that of T1 in a 3/5 to 2/5 ratio, respectively [28]. The “mandibular core line (Md line)” was drawn between two points placed on the inferior surface of the inner contour of the symphysis and the posterior contour of the inferior alveolar nerve (Figure 2D). The mandibular tracings were superimposed on the anterior–inferior contours of the symphysis, just below the pogonion, on the inner contour of the cortical plate at the lower border of the symphysis, and at the posterior contours of the alveolar canal [28].

### 2.4. Linear and Angular Measurements

On the cranial base, maxillary, and mandibular superimpositions of the T1 and T2 cephalograms, the horizontal (x) and vertical (y) distances of the following cephalometric points were measured. The directions of the horizontal and vertical movements were recorded by measuring on a coordinate system. For example, anterior movement is recorded as positive, posterior movement as negative, superior movement as positive, and inferior movement as negative.

The following points were marked on the lateral cephalograms: On a cranial base superimposition, we examined the movements of the nasion, orbitale, porion, A point, and B point. On a maxillary superimposition, we measured the movements of the maxillary first molar, the maxillary central incisor, and the A point. On a mandibular superimposition, we measured the movements of the mandibular first molar, the mandibular central incisor, the condylion, and the B point.

- Nasion (N): the most anterior point of the frontonasal suture;
- Orbitale (Or): the most inferior point of the orbital rim;
- Porion (Po): the most superior point of the external auditory meatus;
- Condylion (Co): the midpoint between the most posterior and superior points of the condyle;
- Gonion (Go): the midpoint between the best fit lines tangent to the posterior and the inferior border of the mandible;
- Gnathion (Gn): the midpoint between the most anterior and inferior points of the symphysis;
- A-point (A): the most concave point along the maxillary dentoalveolus between the anterior nasal spine and the crestal bone;
- B-point (B): the most concave point along the mandibular dentoalveolus between the crestal bone and the pogonion;
- Maxillary first molar (U6): the furcation between the mesiobuccal and distobuccal roots of the maxillary first molar;
- Maxillary central incisor (U1): the incisal edge of the maxillary central incisor;
- Mandibular first molar (L6): the furcation of the mandibular first molar roots;
- Mandibular central incisor (L1): the incisal edge of the mandibular central incisor.

The following distances and angles were measured on the lateral cephalograms:

- SN: the millimeter distance between the sella and the nasion, representing the length of the anterior cranial base;
- Co-Gn: the millimeter distance between the condylion and the gnathion, representing the mandibular length;
- SNA: the angle between a line through the sella to the nasion and a line through the nasion to the A point;
- SNB: the angle between a line through the sella to the nasion and a line through the nasion to the B point;
- ANB: SNA minus SNB, representing the maxilla–mandibular sagittal relationship;
- SN-GoGn: the angle between a line through the sella to the nasion, and a line through the gonion and the gnathion;
- CB line-A: the angle between the cranial base core line and a line through the CB core point (anterior point of the CB core line) and the A point, representing the maxillary sagittal position relative to the cranial base, instead of the SN, and eliminating the influence of the remodeling of the nasion (Figure 3A);
- CB line-B: the angle between the cranial base core line and a line through the CB core point (anterior point of the CB core line) and the B point, representing the mandibular sagittal position relative to the cranial base, instead of the SN, and eliminating the influence of the remodeling of the nasion (Figure 3A);
- A-CB point-B: the difference between CB line-A and CB line-B, representing the maxilla–mandibular sagittal relationship. It is analogous to the ANB, but uses the CB point instead of the nasion, eliminating the influence of the remodeling of the nasion (Figure 3A);
- CB line-GoGn: the angle between the cranial base core line and a line through the gonion and the gnathion. The T2–T1 difference represents the mandibular plane rotation relative to the cranial base (Figure 3B);
- CB line-Mx point: the angle between the cranial base core line and a line through the cranial base and the maxillary core points. The cranial base core point is the anterior point of the cranial base core line. The maxillary core point is the anterior point of the maxillary core line. The T2–T1 difference represents the changes in the maxillary sagittal position (Figure 3C);
- CB line-Md point: the angle between the cranial base core line and a line through the cranial base and the mandibular core points. The mandibular core point is the anterior point of the mandibular core line. The T2–T1 difference represents the changes in the mandibular sagittal position (Figure 3C);
- Mx point-CB line-Md point: the difference between the CB line-Mx point and the CB line-Md point, representing the maxillomandibular relationship (Figure 3C);
- CB line-Mx line: the angle between the cranial base core line and the maxillary core line. The T2–T1 difference represents the maxillary rotation relative to the cranial base (Figure 3D);
- CB line-Md line: the angle between the cranial base core line and the mandibular core line. The T2–T1 difference represents the true mandibular rotation relative to the cranial base (Figure 3D);
- Co-Md line: the angle between the mandibular core line and a line through the posterior end of the mandibular core line and the condylion. The T2–T1 difference represents the direction of the condylar growth relative to the mandibular body (Figure 3E);
- Md line-Md Border: the angle between the mandibular core line and the best fit line to the inferior border of the mandible. The T2–T1 difference represents the degree of remodeling that occurs on the mandibular inferior border (Figure 3F);
- Gonial angle: the angle formed by the junction at the gonion of the posterior border of the ramus and the inferior border of the body of the mandibles.

### 2.5. Statistical Analysis

The means, standard deviations, and ranges were calculated for the resulting data, showing the annual changes between the T1 and T2. A student paired *t*-test was used to confirm the statistical significance between T1 and T2 (*p* < 0.05). Intraexaminer reproducibility was tested by remeasuring 8 randomly selected patients at least one month apart (the same examiner, L.Y.). The intraexaminer error between two times of measurements was determined using a paired *t*-test and the intraclass correlation coefficient (ICC). In addition, the Pearson correlation coefficient was measured to examine the strength between the association of the two variables.

## 3. Results

### 3.1. Cephalometric Point Distance

**Figure 4:**
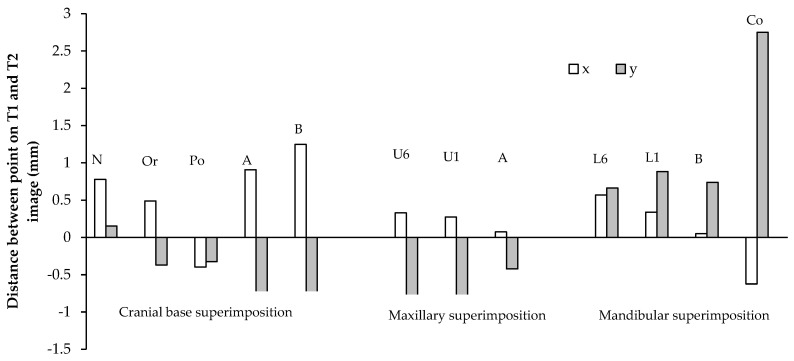
Sagittal (x) and vertical (y) movements of cephalometric points in males (mm/year). The average annual sagittal (x) and vertical (y) movements of cephalometric points on cranial base, maxillary, and mandibular superimpositions of the T1 and T2 lateral cephalograms in males.

**Figure 5:**
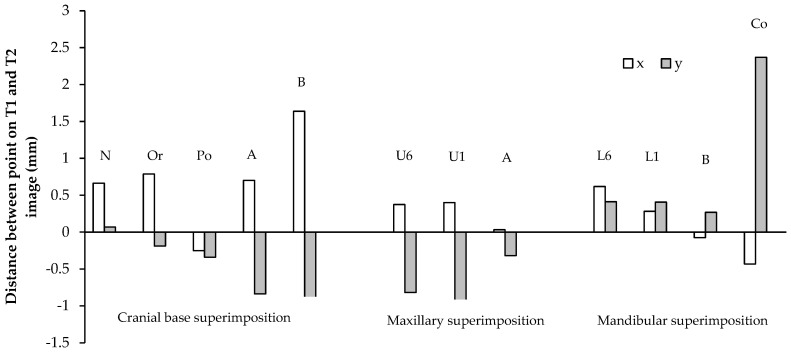
Sagittal (x) and vertical (y) movements of cephalometric points in females (mm/year). The average annual sagittal (x) and vertical (y) movements of cephalometric points on cranial base, maxillary, and mandibular superimpositions of the T1 and T2 lateral cephalograms in females.

The T1 and T2 lateral cephalograms were superimposed on the cranial base, and were oriented so that the Frankfurt horizontal line was parallel to the true horizontal line. The sagittal (x) and vertical (y) components of the distance between the cephalometric points on the T1 and T2 lateral cephalograms were measured, as shown in Table 1, Figure 4 and Figure 5.

All points on the cranial base superimposition moved anteriorly, except for the porion, which moved posteriorly, 0.40 and 0.25 mm/year in males and females, respectively (Table 1). All points moved inferiorly, except for the nasion. The nasion showed a large range of vertical movement in both males and females. The greatest sagittal and vertical movement was seen in the B-point. In males, the B-point moved anteriorly 1.25 mm/year, and inferiorly 0.80 mm/year. In females, the B-point moved anteriorly 1.64 mm/year, and inferiorly 0.90 mm/year. The sagittal and vertical movements of the maxilla were generally less than those of the mandible. In males, the A-point moved anteriorly 0.91 mm/year, and inferiorly 0.98 mm/year. In females, the A-point moved anteriorly 0.70 mm/year, and inferiorly 0.84 mm/year.

On the maxillary and mandibular superimpositions, the A-point and B-point showed a small forward movement in males (*p* > 0.05). In females, both the sagittal and vertical movements of the A- and B-points had a large range, and the averages were statistically insignificant (*p* > 0.05). The U6 and U1 displayed greater vertical dentoalveolar eruption than the L6 and L1 in both genders. The U6 and U1 descended 0.94 and 1.01 mm/year in males, and 0.82 and 0.95 mm/year in females. The L6 and L1 ascended 0.66 and 0.88 mm/year in males, and 0.41 mm/year for both in females. The L6 mesialized more than the U6 in both genders. The L6 and U6 moved anteriorly 0.57 and 0.34 mm/year, respectively, in males, and 0.62 and 0.28 mm/year, respectively, in females. The condylion (Co) demonstrated the greatest vertical movement per year. In males, the condylion moved 2.75 mm/year superiorly, and in females the condylion moved 2.37 mm/year superiorly.

### 3.2. Linear and Angular Changes

**Figure 6:**
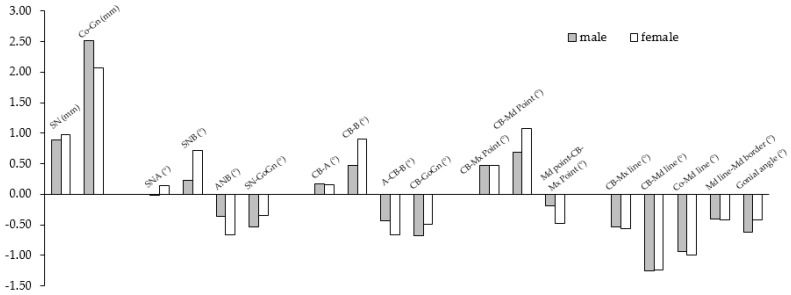
The annual average changes in the linear and angular measurements in males (gray) and females (white).

The angular and linear changes in males and females are shown in Table 2 and Figure 6. The anterior cranial base (SN) increased in length, 0.90 and 0.98 mm/year in males and females, respectively. The mandibular length (Co-Gn) increased in length, 2.51 and 2.06 mm/year in males and females, respectively. This increase was significantly greater in males than females (*p* < 0.05). The SNA, SNB, and ANB displayed very little mean annual change in males. In females, the SNA change was not statistically significant (*p* > 0.05), but the SNB and the ANB showed an annual increase of 0.71 degrees/year, and a decrease of 0.66 degrees/year, respectively. The SN-GoGn displayed an annual decrease of 0.54 mm/year in males (*p* < 0.05), and was not statistically significant in females (*p* > 0.05).

CB line-A and CB line-B assess the maxillary and mandibular positions relative to the cranial base core line, instead of the SN. Both values displayed a very little mean annual change, close to 0 degrees/year in males. In females, CB line-A was not statistically significant, but CB line-B displayed an increase of 0.91 degrees/year. In males and females, A–CB point–B decreased 0.43 and 0.67 degrees/year, respectively.

The CB line-Mx point and the CB line-Md point assess the maxillary and mandibular positions relative to the cranial base core line using the Mx point and the Md point, instead of A and B. Unlike the former measures of the maxillary and mandibular positions and their interrelationship, both showed statistically significant annual increases in both genders (*p* < 0.05). The CB line-Mx point increased 0.47 and 0.48 degrees/year in males and females, respectively, and the CB line-Md point increased 0.69 and 1.08 degrees/year in males and females, respectively. The Mx point-CB line-Md point displayed a decrease of 0.19 and 0.41 degrees/year in males and females, respectively.

The maxillary and mandibular rotations were quantified with the CB line-Mx line and the CB line-Md line. The CB line-Mx line decreased by 0.53 and 0.57 degrees/year in males and females, respectively, which indicates a forward rotation of the maxilla. The CB line-Md line decreased by 1.26 and 1.24 degrees per year in males and females, respectively, which also indicates a forward rotation of the mandible. The mandibular plane relative to the cranial base core line (CB line-GoGn) decreased 0.67 and 0.49 degrees/year in males and females, respectively, indicating a forward rotation of the mandibular plane. The inferior mandibular border (Md line-Md border) remodeled in the opposite backward direction, by 0.41 and 0.43 mm/year in males and females, respectively. The long axis of the condyle was found to move in a forward direction relative to the mandibular core line (Co-Md) by 0.94 and 0.99 mm/year in males and females, respectively. The gonial angle decreased by 0.63 and 0.43 mm/year in males and females, respectively.

### 3.3. Intraexaminer Reliability

The intraexaminer reliability test revealed no statistically significant differences between the data collected from the two times of measurements of the eight randomly selected patients (*p* > 0.05). In each case, the ICCs ranged between 0.90 and 0.99, indicating excellent reliability.

## 4. Discussion

In our study, we have two distinct points, compared with the previous studies. One is that we extracted the lateral cephalograms from the patients’ CBCTs to avoid the conventional limitations of 2D X-rays. Orthogonal images were created to eliminate the magnification error. The right half of the head extending to the left central incisor of the left side was compressed on the CBCT image to improve the visibility of the normally superimposed structures. Therefore, the results of our study convey a more precise depiction of the facial development. Secondly, in addition to the conventional SN reference line, we used the cranial base core line (CB line), based on the anterior cranial base, which is stable over the period. The sella has been reported to exhibit downward and backward remodeling by previous studies [7]. The nasion grew forward in all patients relative to the cranial base, due to a combination of the apposition on the frontal bone, and the enlargement of the frontal sinus [29,30]. In our study, the nasion displayed a large variation in vertical movement, which is consistent with the findings of Bjork and Skieller [2]. Thus, the SN is a not a reliable reference plane for longitudinal comparisons.

The angular measurements using the S, N, A-point, and B-point may not reflect the true annual changes in maxillary and mandibular sagittal development. For example, although the A-point displays significant forward and downward movement on a cranial base superimposition, the difference of the SNA in males and females is close to 0 degree/year, which has been reported by Ranly [30]. The absolute values of change in CB line-A, CB line-B, and CB line-GoGn in our study are more positive than the SNA, SNB, and SN-GoGn, respectively. The CB line-Mx point and the CB line-Md point demonstrate even greater increases, which would be expected, as the maxilla and mandible are known to grow down and forward relative to the cranial base. We found that the cranial base core lines eliminate the uncertainty arising from the continuous remodeling. The angular measurements using the CB-line as a reference line are presumably more accurate tools for a longitudinal comparison of the T2–T1 differences.

Our study shows that the Frankfurt horizontal plane demonstrates questionable reliability as a reference plane, due to the remodeling of the orbitale and the porion. The orbitale exhibits a wide range of vertical change relative to the cranial base, which is due to the variable apposition that occurs as compensation for the descending maxilla [2]. We found the porion displaced inferiorly and posteriorly in most of our subjects. This is expected, as the glenoid fossa, which is also a part of the temporal bone, has been shown to displace inferiorly and posteriorly because of the lengthening and flexure of the posterior cranial base from the developing spheno-occipital synchondrosis [20,31].

In addition, we used the maxillary and mandibular core lines, based on the stable structures in the maxilla and mandible, for our analysis. Relative to the cranial base core line, our study showed that true mandibular and maxillary rotation occurs in a forward (counterclockwise) direction (CB line-Md line: −1.26 and −1.24 degrees/year; CB line-Mx line: −0.53 and −0.57 degrees/year in males and females, respectively). The mandible rotated approximately twice as much as the maxilla, and the rotations were also positively correlated with each other (R = 0.75, *p* < 0.01), which is consistent with the findings of Bjork and Skieller [1], as well as other implant studies [2,31]. Bjork and Skieller [1] also reported that 2 out of 21 patients exhibited the backward rotation of the jaws. In this study, one female displayed backward (clockwise) rotations of the maxilla and mandible. One male displayed a backward rotation of the maxilla, but the mandibular rotation could not be assessed because the patient was not in occlusion. Wang et al. [17] and Spady et al. [32] found true mandibular rotations of about 1.3 and 0.8 degrees per year, respectively, during the primary and early mixed dentition phases.

In our study, we found that the anterior surface of the maxillary corpus is quite stable from T1 to T2 (Table 1). Our findings are in agreement with Bjork [33], and Bjork and Skieller [1], but disagree with Enlow [34,35], who reported this area to be resorptive. Thus, the anterior surface of the maxilla may be used as a stable area for superimposition in untreated growth studies.

The mandibular border’s compensatory remodelings were in the opposite direction of forward rotation (Md line-Md border: −0.41 and −0.43 deg/yr in males and females, respectively), at about a third of the true rotational rate of the mandible, which is consistent with the findings of Wang et al. [17], and is less than what was found by Bjork and Skieller [1], who reported that compensatory remodeling masked about half of the true rotation of the mandible. There was a positive correlation between the true mandibular rotation and the compensatory remodeling of the mandibular inferior border (R = 0.73, *p* < 0.001), which is consistent with previous findings [1,17,32,36]. Spady et al. [32] reported the different rates of true rotation and remodeling depending on the age of the group, which would explain the consistency of this study’s reported rates with that of Lavergne et al. [36], who reported an average over a longer age interval (7–19 years old; 0.9 degree/year true rotation, and 0.5 degree/year of remodeling). Wang et al. [17] suggested that this proportionate increase in compensatory remodeling can be explained by the increased tension from the suprahyoid muscles at the anterior border, causing apposition and compression forces of the masticatory muscle sling, causing resorption at the mandibular angle.

Bjork and Skieller [1] found that the extent of the forward rotation of the jaws was strongly correlated with the forward growth of the condyle and the reduction in the gonial angle. In our study, the condyle appears to rotate forward relative to the mandibular body (Co-Md line: −0.94 and −0.99 deg/year in males and females, respectively), and is positively correlated with true mandibular rotation (R = 0.80, *p* < 0.0001). The gonial angle displayed only a weak-to-moderate correlation with true mandibular rotation (R = 0.48, *p* < 0.05). By contrast, Kim and Nielson found that the intensity of condylar growth significantly varies between subjects, and from year to year in each subject, and that no clear relationship was found between the amount of condylar growth and the mandibular rotation [37].

This study found, from T1 to T2, that the ANB angle reduced by −0.37 and −0.66 degrees per year in males and females, respectively, but this was only statistically significant for females (*p* < 0.05), while previous studies have reported lower rates. The longitudinal study of untreated subjects from the University of Michigan found that the ANB angle decreases 1 to 1.1 degrees between 10 and 15 years of age [38]. Lux et al. found a significant decrease in the ANB angle: from 4.44 to 2.79 degrees among males, and from 3.41 to 2.11 degrees among females, between 7 and 15 years of age [39]. Sassouni reported even lower rates of change of about 0.1 deg/year [40]. This difference possibly comes from the variable age range of the patient population. A-CB point-B and the Mx point-CB point-Md point also show statistically significant decreases in the maxillomandibular relationship, and the latter shows smaller decreases. The difference can only be attributed to the interjaw variation in the location of the A- and B-points.

Our data showed that the condylion grew vertically, 2.75 mm/year in males, and 2.37 mm/year in females, which was consistent with the rate reported in previous studies [21,41]. The mandibular length (Co-Gn) and the vertical growth of the condylion have a positive correlation with the amount of vertical eruption of the upper and lower first molars (R = 0.52, *p* < 0.003 and R = 0.58, *p* < 0.001, respectively). Liu and Buschang [42] reported that dentoalveolar eruption compensates for vertical mandibular growth, and that the two components develop in harmony.

The upper first molar also shows less mesialization (0.33 and 0.37 mm/year in males and females, respectively) than the mandibular first molar (0.57 and 0.62 mm/year in males and females, respectively), likely due to the presence of a larger leeway space in the mandibular arch. The eruption of the upper first molar (−0.94 and −0.82 mm/year in males and females, respectively) is greater than the eruption of the lower first molar (0.66 and 0.41 mm/year in males and females, respectively). While this difference is statistically significant (*p* < 0.05) in females, it was not so in males, likely because of the small sample size.

Previous studies have reported that the maxillary molars and the incisors erupt more than their mandibular counterparts: 1.2 vs. 0.9 mm/yr and 1.0 vs. 0.9 mm/yr, respectively, during adolescence [9,43,44,45,46]. Liu and Buschang [42] report that females 10 to 15 years old exhibited a range of 0.42 mm/year to 0.76 mm/year of mandibular molar eruption, which is consistent with the findings of our study. They also reported that the lower incisors erupted 0.3 to 0.6 mm/year, which is consistent with our findings of 0.88 and 0.41 mm/year in males and females, respectively. The authors [42] found that the rate of dental eruption accelerates before, and decelerates after the age of 11–12 years old. The upper central incisors display a similar rate of vertical eruption: −1.01 and −0.95 mm/year in males and females, respectively, as the upper first molars. This is consistent with the findings of Iseri and Solow [47], who reported that about 5.5 mm of maxillary first molar eruption, and 5 mm of maxillary central incisor eruption occurred in females from the age of 10 to 15 years. Both Iseri and Solow [47], and Bjork and Skieller [1] found that posterior eruption was greater than anterior eruption, and that the upper and lower anterior teeth maintained their inclination relative to the implant lines. In our study, the incisal tips proclined slightly relative to their respective jaws. The discrepancy might come from the difference in accuracy between the 2D and 3D compressed images.

Our study confirms the unique facial patterns and correlations between the developing processes, described by Bjork and Skieller [1,2,3]. Herein lies the limitation of this study: the reported annual changes are an average of multiple facial types, even though the majority of them are skeletal Cl I or II patients with normal vertical mandibular planes. Interestingly, Stahl et al. [48] reported that craniofacial growth in untreated Cl II Division 1 malocclusion is basically similar to that in untreated Cl I subjects at all developmental intervals, except for significantly smaller increases in the mandibular length at the growth spurt (cervical vertebral maturation (CVM) 3–4), and during the overall observation period (CVM1-6). In addition, the amount of counterclockwise rotation of the jaws, the condylar rotation relative to the mandible, and the compensatory remodeling of the mandibular border that was reported for females, are underestimated because of the presence of one patient who was found to be a “clockwise grower”. Because of the small sample size, we could not analyze separately. Future studies with more significant sample sizes would be beneficial to understand the differential growth of the backward and forward rotation of the jaws. In addition, it should be noted that these CBCTs were not taken only for research purposes. Frequent radiation must be avoided (as low as reasonably achievable, (ALARA)), especially in children and for images with an increased amount of radiation. Therefore, the judicious use of computed tomography scanning is essential in order to foster the safest possible care of children.

## 5. Conclusions

1. On a cranial base superimposition, the orbitale, the A-point, and the B-point moved anteriorly and inferiorly. The porion moved posteriorly and inferiorly. The nasion moved inferiorly, and showed a large variation in vertical displacement. The SN and Frankfurt horizontal planes might not be reliable as the reference planes for the longitudinal evaluation of growth;
2. The A-point displayed the inferior movement that is only statistically significant in males, and variable sagittal movement on a maxillary superimposition. The B-point displayed the superior movement that was only statistically significant in males, and variable sagittal movement on a mandibular superimposition. Sagittally, the anterior surfaces of the maxillary and mandibular corpora were stable;
3. Most subjects displayed the forward rotation of the jaws and correlated processes, such as the forward growth of the condyle, the compensatory remodeling of the inferior mandibular border, and the reduction in the gonial angle. These correlated processes occured in the opposite direction for the individual who had backward rotation of the jaws;
4. Males had greater increases in the mandibular length than females. Increases in the mandibular length and the vertical growth of the condyle were correlated with vertical dentoalveolar eruption;
5. The maxillary and mandibular molars and incisors erupted at similar rates. The maxillary molars erupted more than the mandibular molars. The mandibular molar mesialized more than the maxillary molar because of the greater leeway space. The maxillary and mandibular incisors proclined only slightly on maxillary and mandibular superimpositions, respectively;
6. Our limitations of this study are the inclusion of multiple facial types and a small sample size. Future studies with a larger sample size would be beneficial in order to examine the differential growth patterns according to different face types.

## Figures and Tables

**Table 1:** The average annual sagittal (x) and vertical (y) movements of cephalometric points on cranial base, maxillary, and mandibular superimpositions of the T1 and T2 lateral cephalograms (*, *p* < 0.05; NS, not significant).

		Male						Female					
		N	Average	S.D.	Min	Max	*p*−Value	N	Average	S.D.	Min	Max	*p*−Value
Cranial Base													
N	x	11	0.78	0.48	0.00	1.60	*	18	0.66	0.60	0.00	1.87	*
	y	11	0.15	0.46	−0.60	1.26	NS	18	0.07	0.73	−2.23	1.35	NS
Or	x	11	0.49	0.47	0.00	1.54	*	19	0.79	0.54	−0.21	1.76	*
	y	11	−0.37	0.41	−1.37	0.00	*	19	−0.19	0.53	−1.12	0.81	NS
Po	x	11	−0.40	0.33	−0.93	0.16	*	19	−0.25	0.51	−1.33	0.73	*
	y	11	−0.33	0.44	−1.52	0.00	*	19	−0.34	0.46	−1.17	0.49	*
A	x	11	0.91	0.68	−0.56	1.74	*	19	0.70	0.91	−0.95	1.88	*
	y	11	−0.98	0.34	−1.74	−0.49	*	19	−0.84	0.68	−2.29	0.56	*
B	x	7	1.25	1.15	−0.81	2.90	*	13	1.64	0.96	0.00	3.12	*
	y	7	−0.80	1.08	−2.26	0.90	NS	13	−0.90	1.36	−3.03	1.07	*
Maxilla													
U6	x	9	0.33	0.21	0.00	0.54	*	18	0.37	0.37	0.00	1.13	*
	y	9	−0.94	0.61	−1.87	0.00	*	18	−0.82	0.61	−1.74	0.00	*
U1	x	9	0.27	0.24	0.00	0.77	*	18	0.40	0.89	−0.79	2.06	NS
	y	9	−1.01	0.66	−2.44	0.00	*	18	−0.95	1.21	−5.03	0.00	*
A	x	11	0.07	0.43	−0.73	0.77	NS	19	0.03	0.43	−1.28	0.78	NS
	y	11	−0.42	0.47	−1.36	0.00	*	19	−0.32	0.67	−2.36	0.62	NS
Mandible													
L6	x	8	0.57	0.42	0.00	1.16	*	19	0.62	0.49	0.00	1.39	*
	y	8	0.66	0.35	0.00	1.04	*	19	0.41	0.40	0.00	1.40	*
L1	x	8	0.34	0.27	0.00	0.86	*	18	0.28	0.56	−0.85	1.84	*
	y	8	0.88	0.50	0.16	1.54	*	18	0.41	0.45	0.00	1.71	*
B	x	10	0.05	0.11	0.00	0.26	NS	19	−0.08	0.29	−0.79	0.46	NS
	y	10	0.74	0.81	−0.49	1.96	*	19	0.27	1.06	−2.43	2.23	NS
Co	x	11	−0.62	0.51	−1.72	0.00	*	19	−0.43	0.99	−2.47	1.67	NS
	y	11	2.75	0.80	0.73	3.89	*	19	2.37	1.13	0.19	5.15	*

**Table 2:** The annual average changes in the linear and angular measurements in males and females (*, *p* < 0.05; NS, not significant).

	Males						Females					
	N	Average	SD	Min	Max	*p*−Value	N	Average	SD	Min	Max	*p*−Value
SN (mm)	11	0.90	0.49	0.00	1.82	*	18	0.98	0.77	0.00	3.34	*
Co−Gn (mm)	11	2.51	0.51	1.51	3.18	*	19	2.06	0.92	0.50	3.80	*
SNA (°)	11	−0.02	0.77	−2.00	0.95	NS	18	0.15	0.94	−2.12	1.30	NS
SNB (°)	8	0.23	0.49	−0.49	0.84	NS	13	0.71	0.75	−0.64	1.95	*
ANB (°)	8	−0.37	0.53	−1.57	0.11	NS	13	−0.66	0.64	−2.35	0.00	*
SN−GoGn (°)	8	−0.54	0.37	−1.20	0.08	*	13	−0.35	0.86	−1.85	0.57	NS
CB line−A (°)	11	0.16	0.59	−0.85	1.06	NS	19	0.15	0.89	−1.75	1.25	NS
CB line−B (°)	8	0.48	0.61	−0.35	1.48	NS	13	0.91	0.63	−0.29	2.01	*
A−CB point−B (°)	8	−0.43	0.35	−1.13	−0.07	*	13	−0.67	0.69	−2.27	0.03	*
CB line−GoGn (°)	8	−0.67	0.69	−2.17	0.08	*	13	−0.49	0.69	−2.45	0.31	*
CB line−Mx point (°)	11	0.47	0.64	−0.33	1.65	*	19	0.48	0.81	−1.30	1.61	*
CB line−Md point (°)	8	0.69	0.58	0.00	1.65	*	13	1.08	0.73	0.00	2.46	*
Md point−CB point−Mx point (°)	8	−0.19	0.22	−0.65	0.00	*	13	−0.48	0.59	−1.64	0.08	*
CB line−Mx line (°)	11	−0.53	0.47	−1.43	0.10	*	19	−0.57	1.05	−2.87	2.63	NS
CB line−Md line (°)	8	−1.26	0.66	−2.52	−0.47	*	13	−1.24	1.27	−3.90	0.39	*
Co−Md line (°)	11	−0.94	0.68	−1.90	0.10	*	19	−0.99	1.18	−4.41	0.00	*
Md line−Md border (°)	11	−0.41	0.39	−0.84	0.58	*	19	−0.43	0.68	−1.41	1.26	*
Gonial Angle (°)	11	−0.63	0.54	−1.33	0.20	*	19	−0.43	0.77	−2.15	1.02	*

## Data Availability

The data presented in this study are available upon request.
